# Case of Diseased Vena Cava, Terminating in Death by Rupture of the Vein

**Published:** 1846-10

**Authors:** Edward R. Squibb


### MEDICAL EXAMINER

AND

### RECORD OF MEDICAL SCIENCE.

NEW SERIES.—No. XXII.—OCTOBER, 1846.

---

### ORIGINAL COMMUNICATIONS.

*Case of diseased Vena Cava, terminating in death by rupture of the vein.* By Edward R. Squibb, M. D.

On the 30th of April, 1845, I was requested to see a mulatto man of middle stature, somewhat emaciated, aged 37 years, the history of whose illness, as elicited at the time, was briefly as follows :

About nine weeks previous, he had been seized with pain in the abdomen, extending from the lower end of the sternum to the iliac fossa, and chiefly confined to the right side. The patient was a shoemaker, and attributed the attack to sleeping in the cellar where he worked during the day, with his right side to the wall, the neighbouring cellar being at the time half filled with water. The pain was remittent, and increased in severity during several days, when a physician was called in. The nature of the treatment first instituted is not precisely known, though some circumstances render it probable that the affection was mistaken for peritonitis. The course adopted was continued for many weeks without marked improvement, and the case was then abandoned.

*April 30th.* The patient complained of much pain in the abdomen, remittent as before, being increased by taking food or drink, and also during the night. Pressure upon almost any part of the abdomen also caused increase of pain. Stomach irritable, often throwing off whatever was taken into it, giving the patient a sensation of obstruction to the passage of substances taken, as though the outlet of the stomach were tied. Bowels regular; tongue moist, with a slight white fur; appetite bad; pulse 86, somewhat irregular and full, but not hard; skin dry, slightly warmer than natural but not subject to changes of temperature; functions of the liver and kidneys performed apparently as usual; pain sensibly varied by changes of weather. After much indecision and repeated examinations, the case was supposed to be one of rheumatic affection of the intestinal canal, similar to some which have been mentioned by M. Andral.

Twelve ounces of blood were taken by as many cups applied near the spine, and three grains of sulphate of quinia were given every two hours through the day, for four successive days, when the pain was much abated. On the four following days the quinia was given in the same doses, at intervals of four and six hours, in conjunction with a blue pill every morning and evening. The alterative, with an occasional aperient, was continued for many days longer, when the pain, although much abated, was not removed. At this period, a slight exposure, and a change of weather, caused a renewal of all the symptoms, to combat which the same method of treatment was again pursued, but without the same success. A reapplication of the cups did not materially relieve the pain as before, and the quinia produced its peculiar effects upon the senses without much change in the pain. Tincture of colchicum seed was resorted to with no better success, and the mercurial, pushed to the extent of slight ptyalism, was equally without effect. After the suspension of these means, the pain gradually abated as the weather became more favourable, and, when able, the patient was advised to try the effect of change of air and diet. Accordingly, after a treatment of more than two months duration, he left the city for a few months.

On the 3rd of December following, I was called upon to make a post mortem examination in the case, when I found that I had made quite as great a mistake in the diagnosis, as that with which I had mentally charged my predecessor. Sometime after the return of the patient to the city, (he having never recovered sufficiently to permit him to resume his work,) he was again seized with attacks of pain, and called to his aid Dr. J. L. Knight. To the kindness of this gentleman I am indebted for some of the farther particulars, and an opportunity to make a necroscopic examination. After treatment for a day or two, the pain again abated, and the patient felt able to sit up in bed. The exertion of raising up caused a feeling of great weakness and tendency to faint. He was immediately laid down, but expired in the course of a few hours.

Upon turning off the parietes of the abdomen 27 hours after death, the viscera were found imbedded in and quite hidden by masses of coagulated blood, the viscera themselves appearing to be in quite a healthy condition. On seeking for the source of this great effusion of blood, a rupture of the ascending cava was discovered, just below the lower concave surface of the liver. The vein at this point, had been very much dilated, and its coats much diseased and thinned. A semi-organized mass or clot, which was contained in the dilatation, was connected by its surface to the softened coats of the expanded vessel, and the rupture had occurred at the junction of the edge of this mass with the side of the vessel, and not at the projecting point of the dilatation. The tumour was on the anterior portion of the circumference of the vessel, and was overlapped by the lower edge of the liver, and by a portion of the stomach, which latter circumstance may account for the sensation of obstruction so constantly complained of during life.

Although an examination of the heart was desirable, some circumstances prevented an opening of the thoracic cavity, and thus defeated this purpose.

